# Transplantation of Embryonic Spinal Cord Derived Cells Helps to Prevent Muscle Atrophy after Peripheral Nerve Injury

**DOI:** 10.3390/ijms18030511

**Published:** 2017-02-27

**Authors:** Carolin Ruven, Wen Li, Heng Li, Wai-Man Wong, Wutian Wu

**Affiliations:** 1 School of Biomedical Sciences, Li Ka Shing Faculty of Medicine, The University of Hong Kong, 21 Sassoon Road, Hong Kong, China; carolinr@hku.hk (C.R.); liwenhlb@gmail.com (W.L.); lornalih@gmail.com (H.L.); wmwonga@hku.hk (W.-M.W.); 2 State Key Laboratory of Brain and Cognitive Sciences, Li Ka Shing Faculty of Medicine, The University of Hong Kong, Pokfulam, Hong Kong, China; 3 Joint Laboratory for CNS Regeneration, Jinan University and The University of Hong Kong, GHM Institute of CNS Regeneration, Jinan University, Guangzhou 510000, China; 4 Guangdong Engineering Research Center of Stem Cell Storage and Clinical Application, Saliai Stem Cell Science and Technology, Guangzhou 510000, China

**Keywords:** axonal injury, cell transplantation, muscle atrophy, peripheral nerve injury, spinal cord derived cells, fetal neurons, neural progenitor cells, neuromuscular junctions, delayed nerve repair, functional recovery

## Abstract

Injuries to peripheral nerves are frequent in serious traumas and spinal cord injuries. In addition to surgical approaches, other interventions, such as cell transplantation, should be considered to keep the muscles in good condition until the axons regenerate. In this study, E14.5 rat embryonic spinal cord fetal cells and cultured neural progenitor cells from different spinal cord segments were injected into transected musculocutaneous nerve of 200–300 g female Sprague Dawley (SD) rats, and atrophy in biceps brachii was assessed. Both kinds of cells were able to survive, extend their axons towards the muscle and form neuromuscular junctions that were functional in electromyographic studies. As a result, muscle endplates were preserved and atrophy was reduced. Furthermore, we observed that the fetal cells had a better effect in reducing the muscle atrophy compared to the pure neural progenitor cells, whereas lumbar cells were more beneficial compared to thoracic and cervical cells. In addition, fetal lumbar cells were used to supplement six weeks delayed surgical repair after the nerve transection. Cell transplantation helped to preserve the muscle endplates, which in turn lead to earlier functional recovery seen in behavioral test and electromyography. In conclusion, we were able to show that embryonic spinal cord derived cells, especially the lumbar fetal cells, are beneficial in the treatment of peripheral nerve injuries due to their ability to prevent the muscle atrophy.

## 1. Introduction

Peripheral nerve injuries occur frequently and affect 2.8% of trauma patients annually, resulting in loss of motor and sensory functions [1]. Although there are treatments, such as end-to-end repair and conduits for nerve injuries that involve helping the host axons to regenerate and re-establish connections with target muscle, time is still the limiting factor for axon regeneration [2,3]. When the distance from the injury site to the target muscle is too long, for instance, following brachial plexus injury, the axonal regeneration at 1 mm/day [2,4] would take more than a year after injuries of nerves innervating muscles in the lower limbs. During this time, muscles lose the connection with axons, leading to degeneration of muscle endplates and severe muscle atrophy [5]. Muscle atrophy is characterised by a loss in muscle fiber size [6,7,8], appearance of fibrillation potentials [9,10,11,12], decrease in muscle forces [13,14,15] and increased fibrosis [16]. It has been shown that even when the regenerating axons finally reach the muscle, the muscles might not be able to receive the reinnervation and almost never return to their original weight and structure [17,18,19,20]. That is why nerve repair should be done together with some method to prevent muscle atrophy. Currently there is no effective treatment for clinical use to mitigate the muscle atrophy after peripheral nerve injuries.

One of the promising strategies to prevent muscle atrophy is the transplantation of neural or progenitor cells into the distal nerve stump [5]. By selecting a site within the nerve that is in close connection to the target muscle, cells would not have to significantly extend to reach the muscles. The issue of finding the best cells and their source remains. So far, numerous studies have used various kinds of fetal or self-renewal stem/progenitor cells, including embryonic stem cells [21], Schwann cells [22,23], neural progenitor cells (NPCs) [24,25], induced pluripotent stem cells (iPSCs) [26], and stem cells from bone marrow [27,28,29,30], fat tissue [31,32,33], hair [34,35] and skin [36,37,38]. Many of these studies have shown some modest recovery after peripheral nerve injuries [21,30,35,36,38,39,40,41,42,43,44,45,46]. The underlying mechanisms are still unclear and many of the studies focus on speeding up the axonal regeneration rather than preventing the early muscle atrophy. Axonal regeneration can be supported by cells through the production of neurotrophic factors and extracellular matrix components, guidance assistance, remyelination or immune modulation [39]. However, for our objective, (i.e., that is, preventing the muscle atrophy during the regeneration time), the proposed mechanism is mainly through direct neuronal replacement strategy. In this case, motoneurons from cell transplant need to grow towards the muscle fibers and establish new functional neuromuscular connections. However, motoneurons are already mature cells and may not always survive in the central or peripheral nervous system environment and therefore different cell grafts, sources and cell manipulation methods should be studied.

Some previous studies have shown that NPCs are able to survive in the peripheral nerve, differentiate into motoneuron-like cells and reduce muscle atrophy [25,42,47,48,49]. However, NPCs have mostly shown their preferential differentiation into glial lineages [25]. In order to avoid the differentiation concerns, we decided to also use fetal spinal cord cells directly as they have already differentiated into neurons at the isolation time. These few studies that have used fetal spinal cord cells have shown the cells to be able to both assist with the axonal regeneration and with preventing the muscle atrophy [46,50,51,52,53,54,55,56]. Our aim was to compare this kind of fetal cells with pure NPCs for their ability to survive in injured nerve and their ability to reinnervate the muscle and reduce the muscle atrophy. Moreover, we divided the isolated embryonic spinal cords into 3 distinct segments (cervical, thoracic and lumbar) in order to investigate whether the cells from the specific spinal cord segments showed different effects following transplantation into the injured nerve. For the first part of our study, we used adult Sprague Dawley (SD) rats whose right musculocutaneous nerves were transected and above described cells were injected into the proximal nerve stump close to the biceps brachii muscle entering site. Our aim was to identify which cell types are able to survive and prevent muscle atrophy, resulting in an improved muscle condition and better functional neuromuscular connections. In the second part of the study, we used the most beneficial cell type identified, and tested these cells in a more clinically relevant injury model where the nerve was repaired after 6 weeks of delay, to allow the previously degenerated host axons to regenerate.

## 2. Results

### 2.1. In Vitro Characterization of Embryonic Spinal Cord Derived Cells

**Figure 1:**
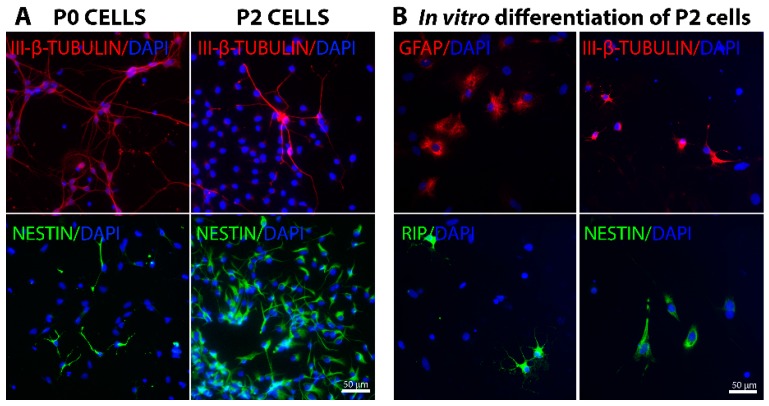
In vitro characterization of cells isolated from E14.5 rat embryo spinal cords. (**A**) P0 cells represent the fetal cells that were directly isolated from embryos and used for transplantation on the same day. Most P0 cells were already of neuronal lineage as shown by their positive III-β-tubulin staining (red). In addition, some Nestin-positive (green) neural progenitor cells were observed. P2 cells represent the cells that were cultured two passages to obtain pure neural progenitor cells (NPCs). Almost all cells at that time were positive for Nestin (green) showing their progenitor cell identity. However, some already differentiated neurons were also seen (red III-β-tubulin staining). DAPI (4′,6-diamidino-2-phenylindole) staining is shown in blue; (**B**) On the 12th day of culturing, some P2 cells were also seeded onto coverslips with “Differentiating medium” for in vitro differentiation experiment. On the 6th day of differentiation, most of the cells had differentiated into GFAP (Glial fibrillary acidic protein)-positive astrocytes (red), with Rip-positive oligodendrocytes (green) and III-β-tubulin-positive neurons (red) also observed. The presence of several Nestin-positive cells (green) indicates undifferentiated immature cells. DAPI staining is shown in blue.

P0 cells are fetal cells that were isolated directly from E14.5 old embryo spinal cord and characterization of these cells showed that around 72%–77% of them were positive for β-III-tubulin showing their neuronal identity (Figure 1A and Figure S1). Nestin-positive progenitors were detected (Figure 1A) at a higher percentage in P0 lumbar cells compared to thoracic and cervical cells (6.5%, 3.6% and 2.1% of cells in the lumbar, thoracic and cervical segment, respectively, Figure S1). This supports the notion that neurogenesis in embryonic spinal cord progresses along the rostral-caudal axis and therefore lumbar segment represent an earlier stage of neurogenesis compared to cervical segment. Furthermore, some Olig2-positive cells, which most likely represent motoneuron progenitors at this stage, were detected (data not shown). Hoxd10 expression was detected only in subtype of neurons in lumbar cell preparation, confirming that we had succeeded isolating correct spinal cord segments (data not shown). For cultured and passaged cells, referred to as P2 cells, we aimed to obtain a pure culture of NPCs by culturing the fetal cells as neurospheres in “NPC medium” that would selectively allow only NPC proliferation. After 2 passages, most of the P2 cells were positive for Nestin showing that we had succeeded in getting the population of NPCs (Figure 1A). However, cell population still contained a small amount of β-III-tubulin positive neurons (Figure 1A), as well as several Rip-positive oligodendrocytes and GFAP (Glial fibrillary acidic protein)-positive astrocytes (data not shown).

Furthermore, we performed in vitro differentiation experiment for 12th day NPCs with “Differentiating medium” that contained serum to facilitate NPCs to differentiate. On the 6th day of differentiation, the cells had mostly differentiated into GFAP-positive astrocytes (around 50%), while a fair number of Rip-positive oligodendrocytes (around 6%), and III-β-tubulin or NeuN positive neurons (around 10%) were also observed (Figure 1B). This proves that these NPCs were able to differentiate into all three lineages, while still most of them preferred the glial lineages. In addition, some cells were still expressing progenitor cell marker Nestin (around 2%) (Figure 1B), showing that they were not yet mature cells.

### 2.2. Grafted Cells Were Able to Survive and Differentiate in the Peripheral Nerve Environment

**Figure 2:**
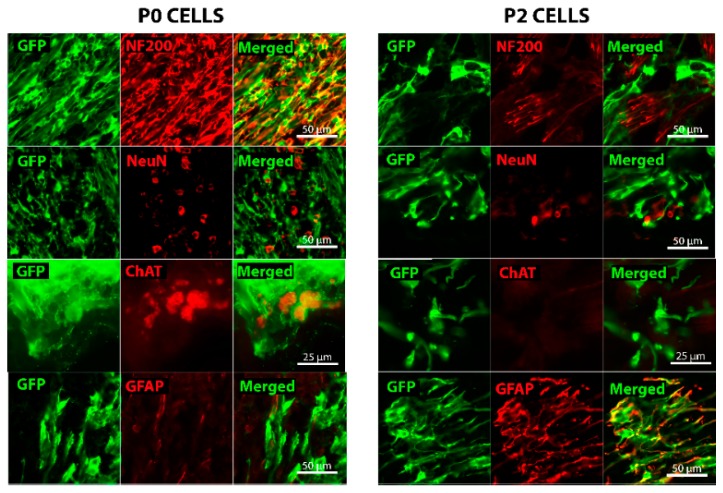
Surviving GFP (Green fluorescent protein)-expressing cells (green) can be seen on longitudinal sections of the musculocutaneous nerve taken from the 6-week time point. The left panels are representative for the P0 cell graft while the right panels for P2 cell graft. Grafted P0 fetal cells seem to have retained their neuronal identity, expressing NF200 (red) and NeuN (red), with some ChAT positive cells (red) detected as well. In contrast, grafted P2 NPCs have mostly differentiated into GFAP (red) expressing astrocytes. However, a few NeuN-positive cells (red) and NF200-positive axons (red) can also be seen even though their GFP expression is really low.

To be able to track the grafted cells, we used cells from transgenic embryos that express GFP (Green fluorescent protein) and therefore all the cells we used were expressing this protein. However, GFP signal had weakened after the transplantation, particularly in differentiated cells. Surviving P0 cells were mostly positive for neurofilament marker NF200 and for markers such as NeuN for neuronal cell bodies while some of the neurons were positive for anti-choline acetyltransferase (ChAT) (Figure 2). Average number of NeuN^+^GFP^+^ cells per field was 122 ± 25 and number of ChAT^+^GFP^+^ cells was 13 ± 2 (Figure S2). This data suggests that P0 cells kept their neuronal identity in vivo. In contrast, P2 cells did not keep their progenitor identity as shown by no positive Nestin staining (data not shown). Instead, the P2 cells mostly differentiated into astrocytes shown by their positive GFAP staining with fewer small NeuN (4 ± 2 cells per field) (Figure S2) and NF200 positive neurons being seen (Figure 2). Very few ChAT^+^ neurons were detected in P2 cell graft (1 ± 0.5 cells per field) (Figure S2).

### 2.3. Muscles from Cell-Grafted Animals Showed Less Atrophy and Larger Muscle Fibers

**Figure 3:**
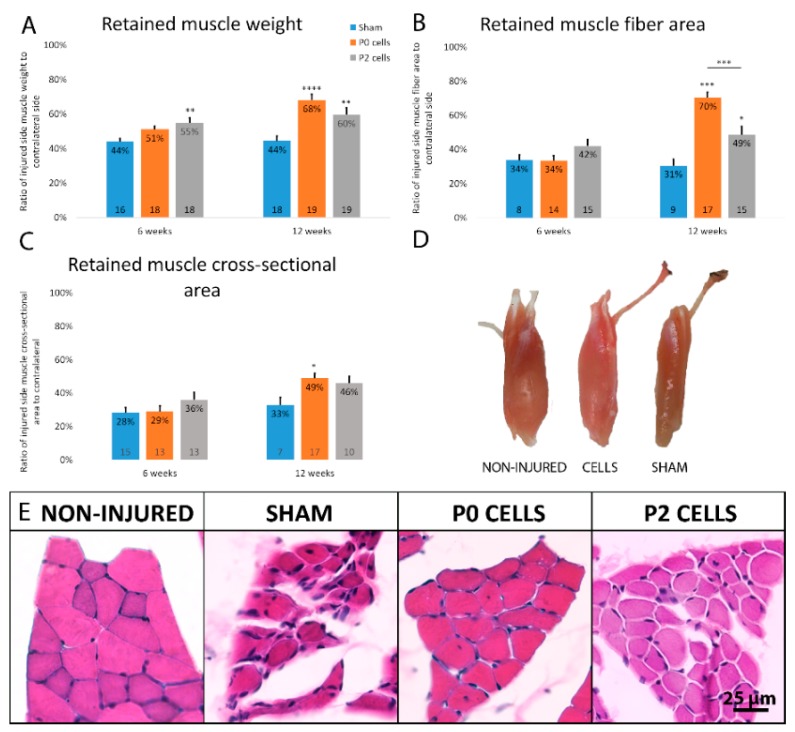
Musculocutaneous nerve transection injury resulted in severe atrophy as seen in the sham animals and also cell-grafted animals after 6 weeks of injury. However, by 12 weeks, cell transplantation into the nerve had helped to reduce the muscle atrophy, shown by regained muscle weight (**A**), regained muscle fiber area (**B**) and regained muscle cross-sectional area (**C**). All the data is expressed as mean + SEM (standard error mean). Statistical difference is shown versus Sham. * *p <* 0.05, ** *p <* 0.01, *** *p <* 0.001. Number of animals in each group is shown inside the bar end; (**D**) Gross morphology of biceps brachii muscle together with the distal part of musculocutaneous nerve 12 weeks after the transection injury of sham, cell-grafted and non-injured contralateral side muscle; (**E**) Hematoxylin-eosin stained muscle cross-sections from 12-week time point show the severe atrophy and smaller fibers in sham muscles and relatively good morphology of muscles from cell-grafted animals.

**Figure 4:**
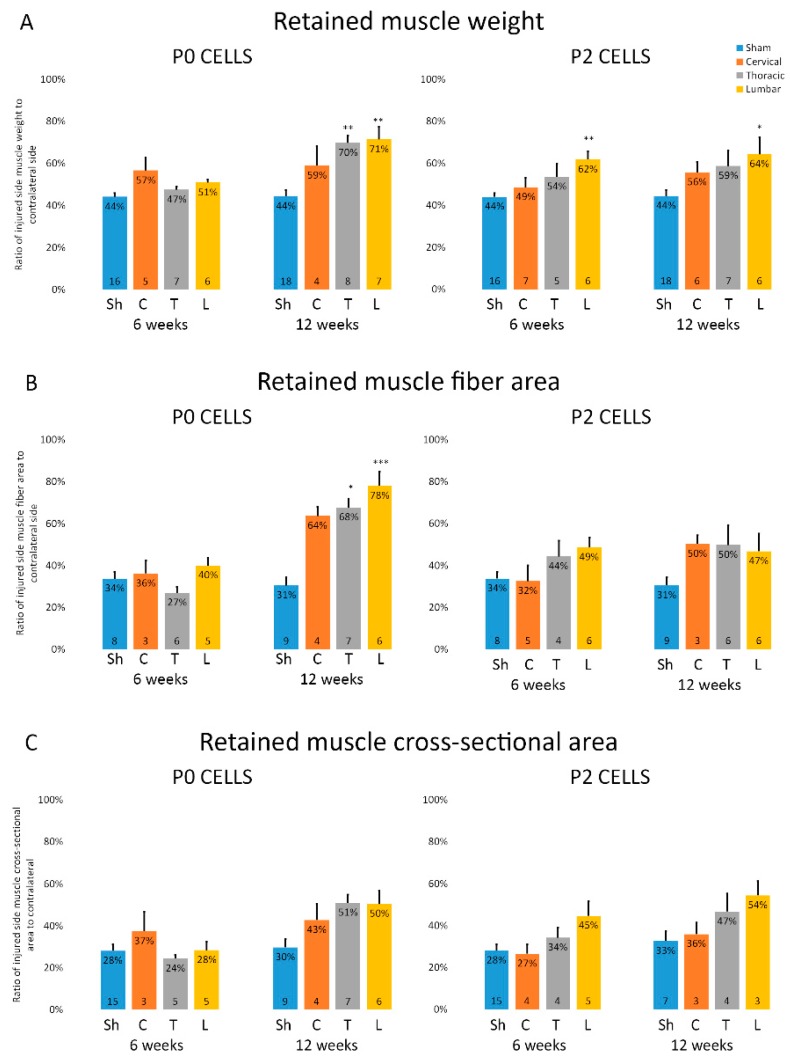
Cells isolated from different embryonic spinal cord segments show differential reduction of muscular atrophy. Sh—sham control, C—cervical cells, T—thoracic cells, L—lumbar cells. (**A**) Analysis of muscle weight (**B**) Analysis of muscle fiber area; (**C**) Analysis of muscle cross-sectional. Data is expressed as mean + SEM. Statistical difference is shown versus Sham. * *p <* 0.05, ** *p <* 0.01, *** *p <* 0.001. The number of animals in each group is shown inside the bar base.

The severity of muscle atrophy that occurs after axonal injury can determine the degree of functional recovery post-reinnervation. In our model, the biceps brachii muscle is innervated only by the musculocutaneous nerve and therefore it undergoes atrophy after this nerve is transected. The muscle wet weight analysis shows that without any treatment, the muscle weight of sham animals was just 42% ± 2% and 43% ± 3% of contralateral side after 6 and 12 weeks, respectively (Figure 3A). In cell-grafted animals, the muscles were bigger than sham ones with visually noticeable differences (Figure 3D). Significant difference between cell-grafted animals and sham animals was already seen after 6 weeks when the retained muscle weight of cell-grafted animals was 51% ± 2% and 55% ± 3% of contralateral side in P0 and P2 cell group, respectively (Figure 3A). After 12 weeks, the difference was even more apparent; retained muscle weight was 69% ± 3% and 60% ± 4% in P0 and P2 cell group, respectively (Figure 3A). A similar phenomenon was seen when measuring the whole muscle cross sections from the same location (Figure 3C). To further assess the muscle atrophy, average muscle fiber size was measured on hematoxylin-eosin stained cross-sections. The muscle fiber area had significantly decreased after injury both in the cell transplantation and in the sham groups (34% ± 3%, 34% ± 3% and 42% ± 4% of contralateral side in sham, P0 and P2 groups, respectively) however no significant difference between these three groups was detected after 6 weeks (Figure 3B). By 12 weeks, the muscle fibers in cell-grafted animals had increased considerably in size, showing the retained muscle fiber area of 70% ± 3% and 49% ± 5% of the contralateral side in P0 and P2 cell group, respectively, as opposed to 31% ± 4% in the sham animals (Figure 3B). As seen from these results, some muscle atrophy still occurs in the early stage, however, in the later stage the transplanted cells stop the atrophy resulting in increased muscle fiber size. On visual examination, the muscles from sham animals had smaller and more disorganized muscle fibers compared to cell-grafted animals, which suggests that the atrophy process was halted by the cell graft (Figure 3E). Interestingly, P0 cells seemed to be more effective in preserving subsequent atrophy compared to P2 cells, even though the difference reached the significant level only in muscle fiber area measurements (Figure 3B). Furthermore, as our aim was also to compare cells from different spinal cord segments, we found that in various muscle atrophy measurements, lumbar cells seemed to prevent muscle atrophy slightly more as compared to cervical and thoracic cells, even though the differences between different spinal cord segments did not reach the significant level (Figure 4A–C).

### 2.4. Distal Nerve of Cell-Grafted Animals Contained a Lot of Small Newly Myelinated Axons

**Figure 5:**
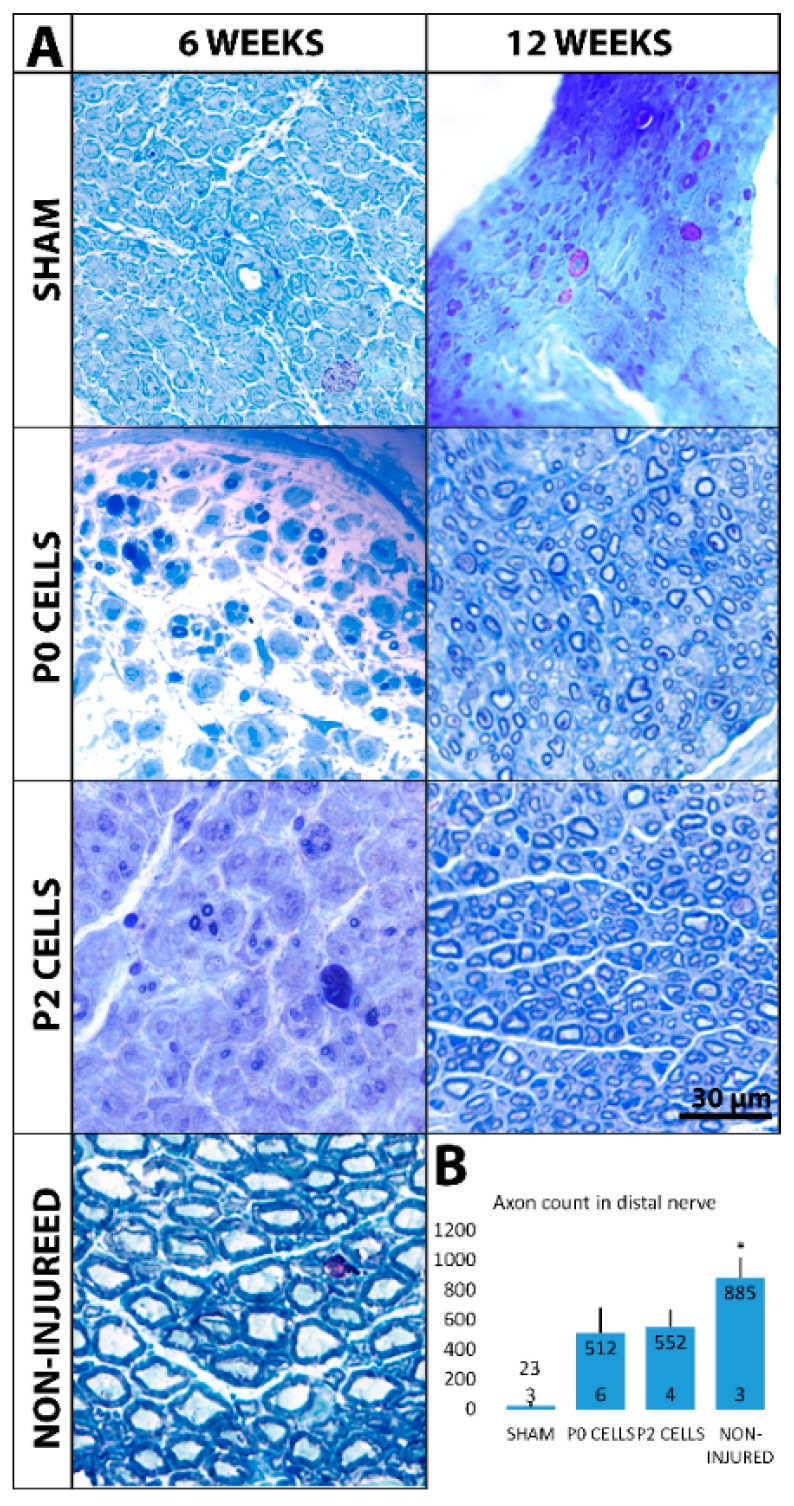
(**A**) Semithin musculocutaneous nerve cross-sections, from sections close to the muscle and distal to the cell graft, stained with toluidine blue show myelinated axons 6 and 12 weeks after the injury. In sham group only very few or no myelinated axons can be seen after 6 and 12 weeks. In cell-grafted nerves, some small, thinly myelinated axons can be seen after 6 weeks, and by 12 weeks, the nerves are fully filled with small thinly myelinated axons. Big myelinated axons from non-injured nerve are shown for comparison; (**B**) The total number of axons in each group is expressed as mean + SEM. Statistical difference is shown versus Sham. * *p <* 0.05. The number of animals in each group is shown inside the bar base.

We next wanted to see if axons from the nerve graft are extending into the muscle. Thick continuous NF200 positive axons were seen in non-injured animals while in sham animals, few discontinuous or even no axons were seen both 6 and 12 weeks after the injury (Figure S3). In contrast, distal nerves from cell-grafted animals showed a lot of NF200-positive axons that were thinner compared to normal axons, suggesting that these axons originated from the grafted cells (Figure S3). Next, Toluidine blue stained semithin nerve cross sections were used to observe myelinated axons. Non-injured nerves showed on average 884 ± 130 big axons (Figure 5B) with a thick myelin sheaths while sham animals showed very few or none myelinated axons both after 6 and 12 weeks (Figure 5A). However, cell-grafted nerves showed a small amount of thinly myelinated small axons after 6 weeks and the number of axons increased very rapidly by 12 weeks when the small, thinly myelinated axons filled all the nerve (Figure 5A), with a total number of 511 ± 165 and 552 ± 106 axons in the P0 and P2 cell groups, respectively (Figure 5B).

### 2.5. Cell Graft Helped to Preserve the Size and Morphology of Muscle Endplates

**Figure 6:**
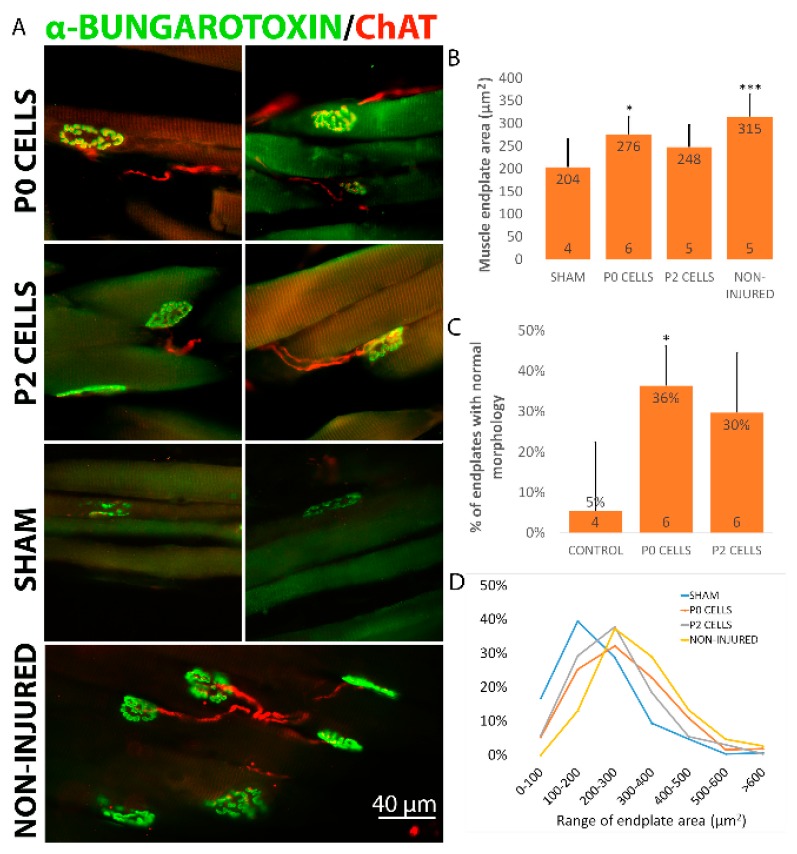
Cell graft derived axons made new connections with muscle endplates and helped to retain their size and morphology. (**A**) Biceps brachii muscle longitudinal sections highlighting neuromuscular junctions, stained with α-bungarotoxin for nicotinic acetylcholine receptors expressed on muscle endplates, and anti-ChAT as an indicator of motoneuron axons. Numerous connections between axons and muscle endplates can be seen on the slides from cell-grated animals, similar to non-injured muscle. In contrast, no axons can be seen making connections with endplates in sham animals and the endplates show shrunken and degenerated morphology. Statistical difference is shown versus Sham; (**B**) Animals from the cell-grafted groups had significantly larger muscle endplates compared to the sham group; (**C**) Numerous endplates in cell-grafted animals had endplates with normal pretzel-like morphology. Data is expressed as mean + SEM. * *p <* 0.05, *** *p <* 0.001. The number of animals in each group is shown inside the bar base; (**D**) Distribution of the muscle endplate areas shows that muscles from sham animals have more endplates with the size of 0–100 and 101–200 µm^2^ compared to the cell-grafted and non-injured groups where more endplates have size around 201–300 and 300–400 µm^2^. All these figures and graphs are shown for 12 weeks timepoint. Statistically significant differences were seen between frequency distributions of different groups (*p <* 0.0001 for all different groups vs. Sham, *p <* 0.0001 for all groups vs. Non-injured, *p <* 0.01 P0 cells vs. P2 cells). All these figures and graphs are shown for 12 weeks timepoint.

The condition of the muscle endplates will determine whether they are able to receive reinnervation from axons and form functional connections for full recovery. In cases where the distance between the injury site and target muscle is long, the degeneration of endplates can be a big problem that leads to unsuccessful reinnervation. Therefore, preserving the normal condition of muscle endplates would help in achieving successful recovery. Our data shows that without any treatment the muscle endplates in sham animals had shrunk in size and showed very irregular morphology being either dispersed or fragmented 6 and 12 weeks after the nerve injury (Figure 6A). Endplates in the sham group were smaller with an average size of 240 ± 19 µm^2^ after 6 weeks and 204 ± 15 µm^2^ after 12 weeks compared to endplates from non-injured side that had an average size of 315 ± 14 µm^2^ (Figure 6B). After 6 weeks, no significant difference was seen in endplate sizes between the sham and cell-grafted animals. However, endplates in cell-grafted animals did not shrink further and by 12 weeks had increased and their average sizes were 276 ± 10 and 248 ± 7 µm^2^ in P0 and P2 cell group, respectively, significantly higher than in the sham animals after 12 weeks (Figure 6B). In addition, the distribution of endplates by size was different in cell-grafted and sham group. Namely, endplates in cell-grafted and non-injured group mostly fall into size category 200–300 µm while the majority of endplates in sham group fall into the 100–200 µm range (Figure 6D). In addition, around 20%–30% and 30%–40% of endplates in cell-grafted groups showed relatively normal pretzel-like structure after 6 and 12 weeks, respectively, while only very few or none pretzel-like endplates were seen in the sham animals (Figure 6C). Next we observed if axons were making connections with the endplates and we were able to detect a considerable number of ChAT-positive axons that reached the α-BTX (α-bungarotoxin)-positive endplates and overlapped with the endplate area (Figure 6A). More precisely, around 60%–70% of endplates in cell-grafted animals were shown to have connections with ChAT^+^ axons. Unexpectedly, some connections were also seen in sham animals, even though there the axons seemed to be discontinuous and the endplates already degenerated (Figure 6A).

### 2.6. Cell-Grafted Animals Showed Functional Neuromuscular Connections

**Figure 7:**
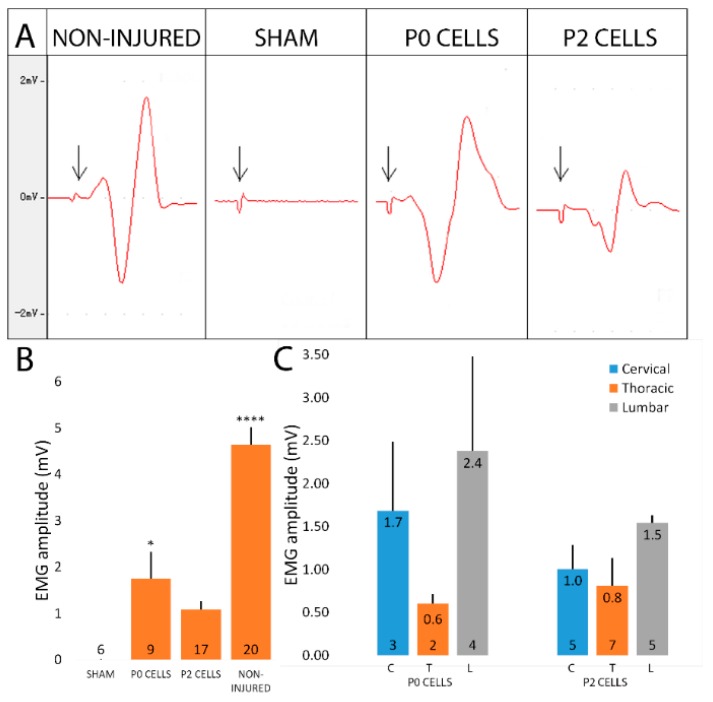
Neuromuscular junctions in cell-grafted animals were functional 12 weeks after the injury and cell transplantation. (**A**) EMG response curves and forelimb movement were similar to the non-injured side in cell-grafted groups while no EMG response and functional improvement was seen in the sham group. The arrow indicates the stimulation artifact; (**B**) P0 cell grafted animals showed higher average EMG amplitude compared to the P2 cell grafted group that were both lower than response amplitude in the non-injured side showing the incomplete reinnervation. As expected, no response was detected from sham animals. Statistical difference is shown versus Sham. Data is expressed as mean + SEM. * *p <* 0.05, **** *p <* 0.0001; (**C**) Animals grafted with cells isolated from spinal cord lumbar or cervical part showed slightly higher EMG response compared to the thoracic cell grafted animals in both P0 and P2 cell groups, though the differences did not reach significant level due to small sample size. The number of animals in each group is shown inside the bar base.

We next asked whether the neuromuscular connections that we saw in cell-grafted animals were also functional and we performed electromyography (EMG) to demonstrate this. When the musculocutaneous nerve in non-injured side was stimulated, successful EMG response with a mean amplitude of 4.6 mV (Figure 7A,B) was easily induced in the biceps brachii, while sham animals showed no response both after 6 or 12 weeks after the injury (Figure 7A,B). This demonstrated that the observed neuromuscular connections in sham animals were not functional, and may have been just some remnants of host connections. In cell transplantation groups, one third of the animals showed response to stimulus after 6 weeks, while after 12 weeks already 60% of animals showed some EMG response. However, the mean response amplitude after 12 weeks was still lower than in normal animals, being 1.8 and 1.1 mV in P0 and P2 cell group, respectively (Figure 7B). Mean response delays were 1.4 ± 0.15 and 1.54 ± 0.16 ms in P0 and P2 groups, respectively, compared to the 0.78 ± 0.03 ms on non-injured side, while sham animals did not have any response and therefore no response delay (data not shown). These results showed that the reinnervation of muscle fibers process is still incomplete by the 12th week. Interestingly, animals with cervical and lumbar cell graft had slightly better EMG responses compared to the animals who received cells from the spinal cord thoracic segment even though the differences were not significant with small sample size. The responses were 1.7, 0.6 and 2.4 mV for cervical, thoracic and lumbar P0 cell group, respectively, and 1.0, 0.8 and 1.5 mV for cervical, thoracic and lumbar P2 cells, respectively (Figure 7C). EMG data supported evidence that the neuromuscular connections seen in the muscles of cell-grafted animals were functional and that successful muscle reinnervation was in progress.

### 2.7. Cell Grafted Animals Show Earlier Functional Recovery after Delayed Nerve Repair

**Figure 8:**
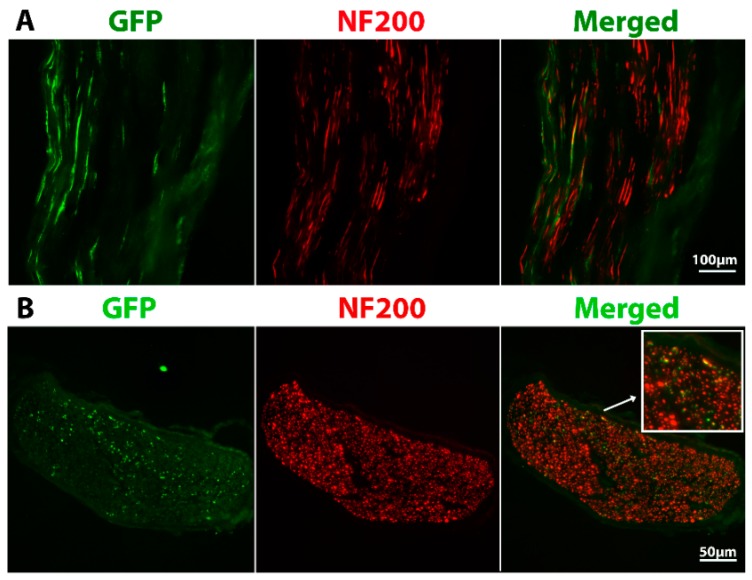

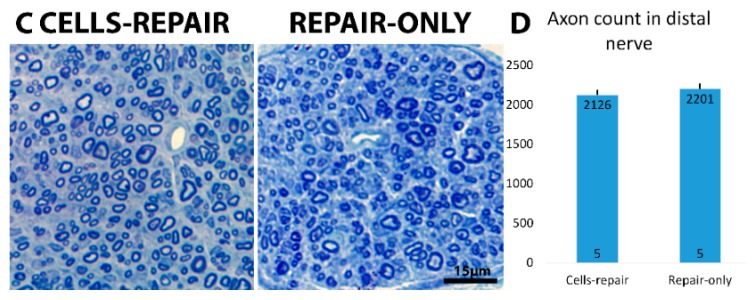
(**A**) Green GFP^+^ axons from the cell graft were seen together with the host regenerating axons in longitudinal musculocutaneous nerve sections; (**B**) Cross sections of the same nerve show the existence of both GFP^+^NF200^+^ (from cell graft) and GFP^−^NF200^+^ axons (regenerating axons from the host); (**C**) The sectioned segment of the nerve was filled with small, thinly myelinated axons in both the cells-repair and the repair-only group seen from semithin nerve cross-sections with Toluidine blue staining; (**D**) These axons were counted and were expressed as a whole number of axons per nerve. No difference was seen between these two groups. Data is shown as mean + SEM. The number of animals in both groups is shown inside the bar base. The number of animals in both groups is shown inside the bar base.

**Figure 9:**
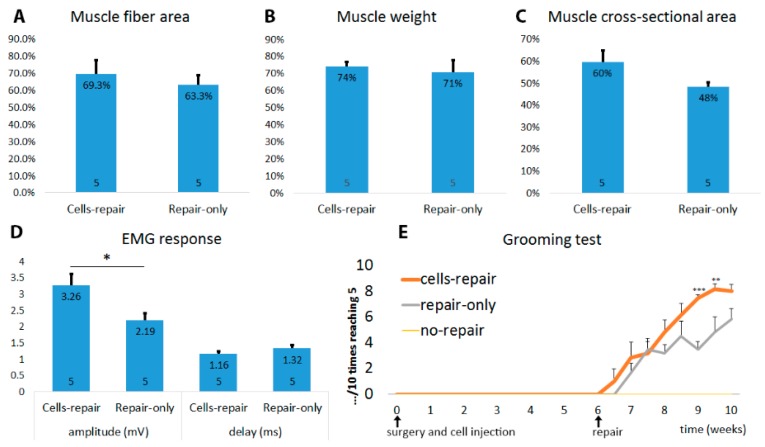
Delayed repair after the injury helped to stop the muscle atrophy, while cells did not significantly increase this positive effect. (**A**) Analysis of muscle fiber area; (**B**) Analysis of muscle weight; (**C**) Analysis of muscle cross-sectional area; (**D**) The muscles in both repair groups were able to evoke EMG response, whereas response in the cells-repair group was significantly higher compared to the repair-only group. Response delay was also slightly improved with cell transplantation. Data is shown as mean + SEM. * *p <* 0.05. The number of animals in each group is shown inside the bar base; (**E**) Grooming test showed that animals without repair did not recover their elbow flexion function while animals who received 6 weeks delayed repair all recovered their elbow function. Interestingly, the cells-repair group showed slightly earlier functional recovery. ** *p <* 0.01, *** *p <* 0.001. Data is shown as mean + SEM. Grooming test results are shown for 6 animals per group.

We were able to show that embryonic spinal cord derived cells are able to prevent the muscle atrophy after the nerve transection and therefore we wanted to test them in a clinically more relevant model. For that intention, we made nerve crushes with 2 ligations to damage the nerve, inhibited regeneration, and waited for 6 weeks by what the axons in distal stump had all regenerated. Immediately after the injury, we injected P0 fetal lumbar cells that were the most successful in previous part of the study into the musculocutaneous nerve close to the muscle. Our aim was to see if these injected cells would help to preserve the muscle endplates during deinnervation and if it would later result in earlier functional recovery after the repair. 6 weeks later we removed the ligations to allow axons from the proximal stump to regenerate into the distal stump. After another 6 weeks, both GFP+ cells and host regenerating axons had extended towards the muscles as seen in the cross and longitudinal sections of the nerve from the distal portion (Figure 8A–C). Approximately 10%–20% of regenerated axons were from the cell graft shown by positive GFP staining (data not shown). During this 6-week regeneration period, twice-weekly grooming test showed that animals receiving cell graft before the delayed repair had significantly earlier functional recovery compared to the repair-only group (Figure 9E). Moreover, the cells-repair group reached almost normal function (score 8–9/10) already by 3 weeks after the repair in contrast to the repair-only group that reached this score by 5.5 weeks after the repair. Even though the behavioral test score at the endpoint of this study did not show any significant difference between the two groups, the importance here is that the cells-repair group recovered much earlier compared to the repair-only group. Improved muscle function was also shown with significantly higher EMG response (3.26 ± 0.31 versus 2.19 ± 0.16 mV in cells-repair and repair-only group, respectively) and shorter response delay (1.16 ± 0.12 versus 1.32 ± 0.06 ms in cells-repair and repair-only group, respectively) in cells-repair group compared to the repair-only group (Figure 9D).

### 2.8. Grafted Cells Help to Preserve the Muscle Endplates until Regenerating Axons Reach the Target

**Figure 10:**
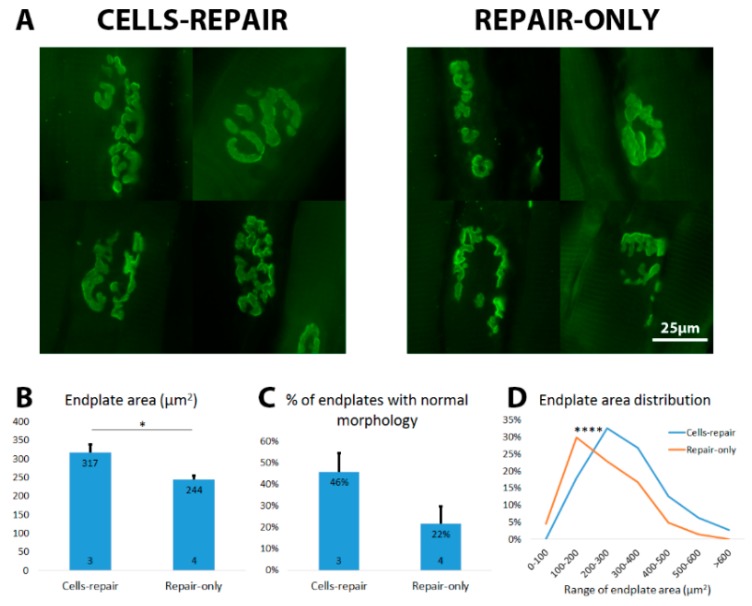
Cell transplantation together with a delayed nerve repair helped to preserve the muscle endplates compared to the repair-only group. (**A**) Typical muscle endplates stained with α-BTX from the both groups are shown; (**B**) The cells-repair group had significantly larger endplates compared to the repair-only group. * *p <* 0.05; (**C**) More endplates with normal pretzel-like morphology were seen in the cells-repair group. Data is shown as mean + SEM. The number of animals in both groups is shown inside the bar base. The number of animals in both groups is shown inside the bar base; (**D**) Distribution of the endplates was shifted to the higher range in the cells-repair group compared to the repair-only group. **** *p <* 0.0001.

Since the cells-repair group showed better functional recovery and EMG response, we proceeded to look at the endplates in the biceps brachii muscle to understand the mechanisms underlying the improved recovery. We noticed that approximately 46% of endplates in the cells-repair group had relatively normal, pretzel-like endplates compared to only around 22% of normal endplates in the repair-only group (Figure 10A,C). Moreover, muscle endplates in the cells-repair group had an average size of 317 µm^2^ while the repair-only group had significantly smaller endplates with average size of 244 µm^2^ (Figure 10B). The distribution of muscle endplates was also shifted to the higher range in the cells-repair group compared to the repair-only group (Figure 10D). Due to the well-preserved muscle endplates, slight prevention of muscle atrophy was also seen in various muscle measurements. Muscle fiber sizes were 69% ± 4% and 63% ± 4% (Figure 9A), wet weights 74% ± 3% and 71% ± 3% (Figure 9B), and whole muscle areas 60% ± 6% and 43% ± 3% (Figure 9C), in cells-repair and repair-only groups, respectively.

## 3. Discussion

### 3.1. Cell Transplantation after Peripheral Nerve Injuries. Which Cells Should Be Used?

Recently, numerous studies have shown that cell transplantation can help with peripheral nerve injuries either speeding up the host axonal regeneration or through direct neuronal replacement strategy to reinnervate the muscles and reduce the atrophy. So far, mostly neural progenitor and embryonic stem cells have shown to act through direct neuronal replacement while other cells, such as stem cells from adipose tissue, hair, skin, bone marrow and amniotic fluid, seem to be beneficial for their regeneration supportive effect through the production of neurotrophic factors, extracellular matrix components, through growth cone guidance and remyelination [30,35,36,38,39,40,41,45]. Promising cells are also iPSCs that have shown the ability to differentiate into motoneurons in vitro [57,58,59] and to form functional connections with reinnervated muscle in vivo [26]. Embryonic fetal cells are safer compared to IPSCs because there is no danger of virus activation or cancer development due to induced pluripotency [60,61,62,63,64,65]. In addition, differentiation potential of iPSCs into specific motoneurons is not well characterized and it is very difficult to obtain fully mature motoneurons from iPSCs [66]. In contrast, fetal embryonic spinal cord cells are already programmed to differentiate into mature and functional motoneurons. In addition, very little work has been done in the field of using iPSCs for nerve regeneration even though iPSCs would be more easily obtained from the same individual [67,68] compared to the NPCs that could be obtained only in small amounts from the adult spinal cord or from electively terminated human embryos [69]. However, self-renewal and neural lineage restricted NPCs in fact have been shown to differentiate into motoneurons that are able to replace the host motoneurons and reinnervate the muscle fibers [25,42,47,48,49]. Different from other studies, our aim was to compare cultured NPCs (P2 cells) with embryonic spinal cord derived fetal cells (P0 cells), as well as comparing cells from different spinal cord segments in their effectiveness for functional recovery. So far, most of the studies before have used NPCs that need to be firstly obtained by culturing the cells with appropriate mitogens. After that, NPCs need to differentiate in the nerve, however the preferential differentiation is towards glial lineages. Few studies in the past that have used isolated fetal spinal cord cells have been shown to be efficient in assisting axonal regeneration and preventing muscle atrophy to a certain degree [46,50,51,52,53]. Taking this into consideration, we decided to use fetal P0 cells that did not need to be cultured and did not need to undergo further differentiation in the nerve. We hypothesized that these P0 cells might be more beneficial for preventing muscle atrophy. Although NPCs might seem to be more promising since they are self-renewing and they proliferate, we believe that the inability of more mature P0 cells to further proliferate does not affect the results when a sufficient number of cells is transplanted. Our results showed that our hypothesis was true as fetal cells had a higher survival rate in the nerve and were able to keep their neuronal identity, whereas the survival rate of NPCs was lower and predominantly produced glial cells rather than neural cells. However, both of the cells were able to extend their axons towards the muscle, form functional neuromuscular junctions (NMJs) and reduce muscle atrophy, although the P0 cells still performed slightly better overall. As such, we believe that P0 fetal cells were better because they did not need to further differentiate into neurons, whereas only a small part of P2 NPCs differentiated into functional neurons as they mostly differentiated into glial cells that could not replace the host axons and could not form new connections with target muscles.

Furthermore, as the spinal cord is divided into many segments based on different functions and morphology, we wanted to see if the cells isolated from either cervical, thoracic or lumbar spinal cord segments are different both in vitro and in vivo. In vivo transplantation experiments showed that in both P0 and P2 cell groups, the cells from lumbar segment were able to prevent the muscle atrophy slightly more than the other cells, though the differences were not statistically significant (Figure 4). That could be explained by the fact that neurogenesis progresses in rostral-caudal axis and therefore the lumbar segment represents the earlier neurogenesis stage, containing more progenitor cells. At the early embryonic stage, these neurons are more immature and probably less sensitive to cell dissociation protocols, which in turn will lead to better survival and beneficial effect in in vivo studies. In addition, both lumbar and cervical cells showed slightly better improvement in EMG responses compared to thoracic cells (Figure 7C). This could be explained by the fact that normally the big motoneurons innervating upper and lower limb skeletal muscles arise from cervical and lumbar segments, respectively, and neurons from thoracic segment do not innervate any skeletal muscles.

### 3.2. Do Fetal Spinal Cord Cells and NPCs Reduce Muscle Atrophy through Direct Neuronal Replacement or Can It Be the Environmental Support?

The first, and most likely, theory to explain our results is that axons from injected cells were able to replace the host axons and form new functional connections with muscles to slow down the progress of atrophy. We showed that transplanted cells were able to survive in the transected peripheral nerve and in both cell groups, numerous surviving cells had extended their axons along the nerve and these thinly myelinated small axons had reached the very distal part of the nerve by 12 weeks filling in the entire nerve trunk. As seen from NMJs staining, some of these axons had already reached the muscle endplates by 6 and by 12 weeks, most of the endplates with normal pretzel-like morphology seemed to be reinnervated. To further prove that the connections we observed in the cell transplantation groups were functional and that these few seen in the sham group were just remnants, we performed EMG studies. As our results showed, the newly formed connections in the treatment groups were able to evoke the EMG response with visible hand movement while no responses were seen in any sham animals, demonstrating that their connection remnants were not functional.

As our aim was to show that cells transplanted into the injured nerve can reduce the muscle atrophy, we used different ways to measure and analyze the severity of muscle atrophy. As expected, muscle atrophy in the sham group was quite severe by 6 weeks (42% ± 4%) and interestingly, the muscle weight did not drop any further by 12 weeks (43% ± 6%) (Figure 3A). The same tendency was observed in the muscle fiber area and cross-sectional area where there wasn’t much difference in retained area between the 6- and 12-week periods in the sham group (approximately 30% of contralateral side in all groups, Figure 3B,C). It could have been assumed that muscles atrophy progressively through the time, but it is likely that the muscles actually atrophy really rapidly right after their loss of innervation and in later stages the atrophy process slows down. Interestingly, severe muscle atrophy and endplate degeneration had also occurred in cell transplantation groups by 6 weeks, but by 12 weeks, grafted cells had reached the target and reinnervated the muscle endplates, thereby helping the muscles to regain approximately 40% and 20% of their fiber size, in P0 and P2 cell groups, respectively (Figure 3B). Since muscles regained their fiber size, it can also be considered as a muscle hypertrophy process. Cell transplantation might have contributed to the muscle hypertrophy in different ways. Firstly, we showed that grafted cells formed new connections with the muscles, restoring the neuromotor activity. In fact, loss of neuromotor activity has shown to be responsible for most of the changes happening during the muscle atrophy [70,71] and therefore restoring the muscle innervation very likely contributes to the muscle recovery and hypertrophy following the atrophy. Secondly, grafted cells produce different neurotrophic factors (NGF, BDNF, GDNF) [25] that might have had a positive effect on muscle atrophy [72]. For example, nerve growth factor (NGF) has shown to play the key role for satellite cell differentiation in muscle repair [73,74]. Similarly, brain-derived neurotrophic factor (BDNF) has shown to regulate satellite cell differentiation and skeletal muscle regeneration [75], while glial-derived neurotrophic factor (GDNF) induces sprouting and muscle fiber reinnervation [76]. This shows that muscle atrophy was being inhibited and reversed from the time when grafted cells had extended long enough towards the muscle to form new NMJs.

Our above stated belief that grafted cells helped the muscles by directly replacing the original host axons and making the new connections with target muscles might not be the only possible explanation to our seen phenomenon. As there remains several unanswered questions in this first theory, then an alternate theory with its pros and cons should be considered; in light of the aforementioned results we now consider if the transplanted cells did not replace the host axons and did not form new connections, they may have just helped the remnants of host axons to survive. This environmental support could be mainly attributed to the production of neurotrophic factors or extracellular matrix components. One argument in support of this idea is that despite being able to see GFP-expressing cells in graft, the axons that further reached the muscle did not always express GFP and could have therefore just been remaining host axons. However, we believe that the GFP expression had simply weakened considerably so that we were not able to detect it in all of the axons and distinguish it from the background noise. In addition, most of the cells in graft that were positive for neuronal cell body markers, and therefore cannot be from the host since there should not be any host neuronal cell bodies in the nerve, had weaker GFP expression compared to the other cells in the cell graft. This interesting phenomenon has been also seen in previous studies where the cell graft derived big motoneurons had very weak GFP expression [25]. In addition, it can be proposed that the GFP expression weakens mostly in cells that differentiated into neural lineage since glial cells in P2 graft showed the GFP expression while few seen neuronal cells did show weak GFP.

Counterarguments for the second theory are as follows: firstly, all these axons in cell-grafted nerves were small in diameter and thinly myelinated in contrast to axons in non-injured nerves that have much larger diameters and thicker myelin sheaths. In the event that the cell graft would have helped host axons to survive, then most likely the axons would have still stayed bigger; secondly, we observed that muscles had atrophied and endplates had degenerated by 6 weeks similarly in cell grafted and sham group, and the differences predominantly appeared at 12-week time point where the muscles in cell-grafted animals had regained their fiber size, weight and endplate size (Figure 3 and Figure 6). This phenomenon is easily explained by the first theory since it takes some time until the grafted cells adapt and extend towards the muscles to reinnervate them. Therefore, the initial atrophy and degeneration could be seen. However, if grafted cells only aided with the survival of host axons then it does not explain why the muscles firstly lost their weight and functionality and then started to regain it at a later stage, as the environmental support from cells by producing neurotrophic or other factors should have expressed an effect in the early stages.

### 3.3. Is It Clinically Relevant?

In our study, we injured the musculocutaneous nerve that arises from the brachial plexus and innervates the biceps brachii muscle. We chose this model because injuries at the brachial level are more common in humans, and brachial nerves have their targets in arms and therefore the damages to these nerves will result in the full or partial loss of motor function of the arm [77]. Our injury model for the main part of this study was chosen to be musculocutaneous nerve transection where nerve stumps were both ligated and kept separately to prevent any regeneration. Additionally, different cells were injected into the distal stump connected to the biceps brachii muscle. We chose not to do any repair in order to avoid the variation in outcome that comes from the host degenerating axons and to focus primarily on the effect of different cells to the muscle atrophy in the absence of externally sourced axons provided. After we identified the best source of cells for prevention of muscle atrophy (i.e., P0 fetal lumbar cells), we decided to test them out on a clinically more relevant injury model. Since in clinic, the aim is still regaining the function of the nerve, then the connection between CNS and PNS has to be reestablished. As a surgical repair, nerve ends can be sutured back together if they are separated after an injury or obstructions can be removed if nerve has been just temporarily crushed [2,3]. Therefore, in the second part of our study, we chose the injury model where crushes and two tight ligations were made on the nerve to temporarily prevent any regeneration and ligations were removed 6 weeks later to allow the delayed regeneration. Since surgical repair is often delayed in clinic, and regeneration progress is slow and distances long, cell injection to the distal nerve is a good potential technique to preserve the target muscles and their ability to later receive host regenerating axons. This part of our study showed that using our most successful P0 fetal lumbar cells was beneficial in preserving the muscle endplates during deinnervation that in turn resulted in earlier functional recovery following host axonal regeneration proceeding delayed repair. Interestingly, we observed that cell transplantation without any repair in our first part of the study was able to preserve the muscle and endplates almost as much as the surgical repair while surgical repair together with the cells was the most beneficial for the overall muscle health and the functional outcome. However, there is a question as to how these host regenerating axons reinnervate the muscle endplates that were already occupied by the grafted cells. It has been previously shown that during regeneration, many of the endplates would be polyinnervated [6,78,79,80]. In the later stage, endplate elimination takes place such that regenerating axons should be able to replace all the grafted cells. This can occur because they receive signals from the spinal cord and they are synaptically more active helping them to win the synaptical competition [81,82,83]. Based on these results, we believe that cell therapy should be considered a potential therapeutic strategy to prevent muscle atrophy after peripheral nerve injuries.

## 4. Materials and Methods

All procedures involving the usage of live animals were approved by the Committee for the Use of Live Animals in Teaching and Research at the University of Hong Kong (CULATR 3167-13, approved on 20 November 2013). The animals were ordered from Laboratory Animal Unit accredited by AAALAC International.

### 4.1. Cell Isolation and Culture

Spinal cords from embryonic day 14.5 rats expressing green fluorescent protein (GFP, SD-Tg(CAG-EGFP)CZ-004Osb “green rat” strain from SLC, Shizuoka, Japan) were isolated out and prepared for cell culture as previously described [25]. Briefly, spinal cords were separated from the surrounding connective tissue and the meninges were peeled off; and the cervical, thoracic and lumbar segments of spinal cord were isolated and transferred separately into 15-mL centrifuge tubes containing HBSS, and centrifuged at 700 rpm for 3 min. Tissues were washed with Hanks’ balanced salt solution (HBSS) and 1 mL of 0.15% Trypsin (Invitrogen, Carlsbad, CA, USA) was added to each tube and incubated at 37 °C for 7 min. 0.5 mL of trypsin inhibitor (10 mg/mL soybean powder in HBSS, Gibco, Waltham, MA, USA) was added to stop the trypsin reaction before the cells were spun down at 1400 rpm for 3 min.

Cells that were to be used for transplantation on the same day (defined as passage zero (P0) fetal cells) were suspended in Dulbecco’s modified Eagle’s medium (DMEM/F12) without supplements to achieve a final concentration of 10^5^ cells/µL. The rest of the cells were transferred into T25 flasks containing “NPC medium” (DMEM/F12, 2% B27, 1% N2, 1% NEAA, 20 ng/mL bFGF, 2 µg/mL Heparin and 100 µg/mL Kanamycin, all from Invitrogen) and cultured as neurospheres. Cells were maintained in a humidified incubator containing 5% CO_2_ at 37 °C. The medium was changed every 2 days and cells were passaged on the 4th and 8th day. On the 12th day of culturing, these neural progenitor cells were prepared for either transplantation or characterization and defined as the second passage (P2) cells.

### 4.2. In Vitro Immunocytochemistry

For in vitro cell characterization of P0 cells, some P0 cells were seeded onto coverslips with “Neural medium” (DMEM/F12, 2% B27, 1% N2, 1% NEAA, all from Invitrogen) immediately following spinal cord dissociation into single-cell culture. Cultured NPCs (P2 cells) were seeded onto laminin and Poly-DL-ornithine (both Sigma-Aldrich, St. Louis, MO, USA) coated coverslips with “NPC medium” on the 12th day of culturing. These cells were used to characterize P2 cells. Both P0 and P2 cells were incubated overnight on coverslips before the cells were fixed in 4% paraformaldehyde (PFA) dissolved in 0.1 M PB (phosphate buffer) for 10 min and washed with PBS (phosphate buffered saline) several times. The following primary antibodies in 1% bovine serum albumin, 0.1% Triton X-100 in PBS were used to stain the cells: mouse anti-Nestin antibody (1:1000, BD Pharmingen, San Jose, CA, USA) for undifferentiated NPCs, mouse anti-β-III-tubulin (1:1000, Sigma-Aldrich) for neurons, rabbit anti-Olig2 (1:1000, Merck-Millipore, Billerica, MA, USA) for motoneuron and oligodendrocyte progenitors, and rabbit anti-Hoxd10 (1:5000, Abcam, Cambridge, UK) for identifying subtype of cells from lumbar segment. The cells were incubated with primary antibodies overnight at 4 °C. Primary antibodies were visualized by incubating with species-specific secondary antibodies conjugated to the fluorescent label Alexa 568 or 488 (1:400, Invitrogen) for 1 h at room temperature. The cells were counterstained with DAPI for nuclei and mounted in antifade mounting medium (Dako, Glostrup, Denmark). The images were taken with a fluorescent microscope (Zeiss Axioplan, Zeiss, Oberkochen, Germany). For quantification, two independent cell batches were used and at least 10 random fields per batch with the magnification of 20× from a coverslip were imaged and positive signals were counted using ImageJ 1.47v and Photoshop CC 2014.

On the 12th day of culturing, some NPCs were also seeded onto coverslips with “Differentiating medium” (DMEM/F12, 1% Fetal Bovine Serum, 2% B27 and 100 µg/mL Kanamycin, all from Invitrogen) to see if these cultured NPCs are able to differentiate into all different neural lineages. The same medium was added every 2nd day and the cells on coverslips were fixed on the 6th day and used for immunocytochemical staining according to the above mentioned protocol. The following primary antibodies were used: mouse anti-Nestin antibody (1:1000, BD Pharmingen) for undifferentiated NPCs; rabbit anti-glial fibrillary acidic protein (GFAP) (1:1000, Sigma-Aldrich) for astrocytes; mouse anti-Rip for oligodendrocytes (1:200, Merck-Millipore) and rabbit anti-β-III-tubulin (1:1000, Covance, Princeton, NJ, USA) for neurons.

### 4.3. Animal Surgery and Cell Transplantation

A total of 112 adult female SD rats (220–250 g) were used for the first in vivo part of the study. Animals were anesthetized with an intraperitoneal injection of ketamine (80 mg/kg) and xylazine (10 mg/kg). A cut was made to the right thorax to expose the musculocutaneous nerve. The nerve was initially ligated with silk sutures at two locations (1 and 1.5 cm from the entry of the nerve into the muscle). Animals were divided into 7 groups (16 animals in each group) receiving either P0 cervical, P0 thoracic, P0 lumbar, P2 cervical, P2 thoracic, P2 lumbar cells or medium (DMEM/F12) injection. One microliter of cells (10^5^ cells/µL) or medium (sham group) was slowly injected into the nerve through a Hamilton syringe (needle gauge 30) distal to the ligations and close to the site where the nerve enters the biceps brachii muscle. After the injection, the musculocutaneous nerve was transected between the two ligations and the nerve stumps were kept far apart from each other to prevent any kind of regeneration. The muscles and fascia were sutured with 5-0 sutures and the skin with 3-0 Ethilon nylon sutures. Antibiotics and analgesics were used for post-operative health care. Animals were allowed to survive for 6 or 12 weeks after the surgery (8 animals in each group).

Another 10 SD rats were used for the next part of the study. In this group, animals were opened and their right side musculocutaneous nerve was crushed and ligated with 2 silk ligations 3 mm apart. Ligations were made strongly to prevent any regeneration and left there for 6 weeks. 1 µL of P0 fetal lumbar cells (10^5^ cells/µL) (cells-repair group) or DMEM/F12 medium (repair-only group) (5 animals per group) was injected distal to the ligations close to the muscle. Animals were closed as described previously. 6 weeks later, the injury site was opened again and repair by removing 2 ligations from the nerve to allow the regeneration was performed. Following this, animals were allowed to survive 6 more weeks.

### 4.4. Behavioral Test

The Terzis grooming test [84] was performed on 10 animals that went through the delayed repair surgery. The test was performed and videorecorded twice a week after the repair surgery. Details about the common test scoring are found in Appendix A. As most of the animals reached the score 4 immediately following the induced injury, the test was scored through a modified system where recovery from score 4 to 5 could be more easily detected. More specifically, we observed the animals doing the grooming movement where non-injured forelimb reached behind the ear 10 times, and counted how many times out of these 10 the injured limb also reached behind the ear and pushed the ear down strongly (score 5). We expressed the results as a number of successful attempts reaching score 5 out of 10 times.

### 4.5. Electromyographic Measurements (EMG)

At the end of the survival time, the animals were deeply anesthetized with an intraperitoneal injection of ketamine (80 mg/kg) and xylazine (10 mg/kg). Their right musculocutaneous nerve and biceps brachii were exposed. A stimulating bipolar electrode was placed under the transected musculocutaneous nerve, and two recording electrodes were inserted into the middle of the muscle belly of the biceps brachii. At least 3 different locations in the biceps were chosen and the forelimb movement and response amplitude were recorded. The same positive stimulating current (1 V, 0.2 ms) was used in all animals. EMG signals were collected with a multi-channel signal acquisition and processing system (RM6240BD, Chengdu, China). The mean EMG amplitude for each experimental group was calculated as an average of the highest amplitudes in each animal with the response and response delay at maximum response amplitude was measured and is shown as the average delay. 20 animals were also opened from the left non-injured side to record the normal baseline EMG response and delay for the same stimulus that are expressed as an average.

### 4.6. Tissue Processing

Immediately after the EMG measurements, anesthetized animals were perfused intracardially with 0.9% saline followed by 4% paraformaldehyde (PFA) solution in 0.1 M PB. Both left and right musculocutaneous nerve and biceps brachii muscle were harvested and the muscles were weighted on laboratory scale. The percentage of retained muscle weight was calculated for each animal by dividing the wet weight of right side muscle with the weight of left non-injured side muscle. After weighting, the tissues were postfixed in 4% PFA solution overnight and stored in 30% sucrose in 0.1 M PB solution at 4 °C for at least 3–4 days. The nerve and muscle tissues were cut into 8–10 µm thick frozen sections using a Leica CM1980 cryostat for immunohistochemical and hematoxylin-eosin staining.

### 4.7. Immunohistochemistry

Longitudinal (8–10 µm thick) nerve sections were washed in 0.01M PBS, blocked in dilution buffer containing 5% normal goat or donkey serum, 2% of BSA and 0.03% of Triton-X-100 in 0.01 M PBS for half an hour at room temperature, followed by antigen retrieval by incubation with proteinase K (10 µg/mL, Invitrogen) for 10 min at 37 °C and finally incubated with primary antibody in dilution buffer at 4 °C overnight. The following primary antibodies were used: mouse and rabbit anti-NF200 (1:500, Sigma-Aldrich) for neurofilaments, mouse and rabbit anti-β-III-tubulin (1:1000, Covance) for neurons; mouse anti-NeuN (1:1000, Merck-Millipore) for neuronal nuclei; rabbit anti-GFAP (1:1000, Sigma-Aldrich) for astrocytes; and mouse and rabbit anti-GFP (1:500, Invitrogen) to intensify the signal from cell graft. Primary antibodies were visualized by incubating with species-specific secondary antibodies conjugated with the fluorescent label Alexa 568 or 488 (1:400, Invitrogen) for 2 h at room temperature. Sections were counterstained with DAPI for 5 min at room temperature and mounted in antifade mounting medium (Dako). The slides were observed under a fluorescent microscope (Zeiss Axioplan). Positive and negative controls were used to validate the staining. Positive controls used were non-injured nerve for NF200 staining and spinal cord for ChAT, GFAP and NeuN stainings. In some slides, primary antibody was omitted in IHC staining to serve as a negative control. In longitudinal nerve sections that contained GFP^+^ grafted cells, quantification for NeuN and ChAT was done. NeuN^+^GFP^+^ and ChAT^+^GFP^+^ cells were counted on 5 or more fields per animal and averaged for 5–6 animals per group. 40× magnification was used and fields with similar amount of total GFP^+^ cells were used for counting with the help of Adobe Photoshop CC.

### 4.8. Examination of Neuromuscular Junctions (NMJs) and Endplates

8 µm thick longitudinal sections of the biceps brachii muscle belly were stained with goat ChAT primary antibody (1:80, Merck-Millipore) to visualize the motoneuron axons. After overnight incubation with primary antibody, slides were incubated with appropriate secondary antibody (Alexa 568, Invitrogen) for 2 h at room temperature, followed by 30 min at room temperature incubation with Alexa 488-labelled α-BTX (1:500, Invitrogen) to visualize postsynaptic acetylcholine receptors of the muscle endplates. The slides were observed under fluorescent illumination using a Zeiss Axioplan microscope. In each muscle, the areas of at least 50 endplates were measured and expressed as the average endplate area. Furthermore, percentage of endplates in each group falling into specific size range (<100, 101–200, 201–300, 301–400, 401–500, 501–600 and <601 µm^2^), were calculated and distribution curves were drawn. Based on the endplates’ morphology, they were divided into endplates with normal or abnormal morphology, and the percentage of endplates with normal morphology was calculated. Normal morphology was considered to be pretzel-like and with a lot of grooves like in non-injured muscle while the abnormal morphology included endplates with fragmented, shrunken or dispersed appearance. Furthermore, number of endplates with obvious connection between the endplate and neuronal axon were counted and are shown as a percentage to all endplates.

### 4.9. Assessment of Myelinated Axons

The distal part of musculocutaneous nerves from 3–6 animals in each group were fixed in 2% PFA and 2.5% glutaraldehyde in 0.1 M PB at 4 °C overnight, postfixed in 1% osmium tertroxide at 4 °C for an overnight, dehydrated in serial concentrations of ethanol, infiltrated with propylene oxide, and embedded in Epon. Semithin (0.5 µm) nerve cross sections distal from the cell graft were cut using a microtome (Leica Ultracut, Leica, Wetzlar, Germany), and stained with 0.5% toluidine blue in 1% borax for light microscopy (Zeiss Axioplan) to identify myelinated axons.

### 4.10. Histological Assessment and Muscle Fiber Measurements

Cross sections from the mid belly of the biceps brachii (7 mm from the muscle bottom) were stained with hematoxylin and eosin to analyze muscle morphology and to measure muscle fiber areas. Stained slides were observed under a light microscope (Zeiss Axioplan) and pictures with 20× and 40× magnification were taken for muscle fiber measurements. In each muscle, the individual area of at least 500 muscle fibers across the muscle was measured and the mean was calculated. Results are expressed as percentages calculated by dividing the mean muscle fiber area (µm^2^) of injured side muscle with the contralateral side muscle fiber area (µm^2^). Furthermore, pictures of muscle cross sections before the staining were taken with magnification 5× and stitched for the whole muscle area measurement. Whole cross sectional area (mm^2^) of biceps brachii long head was measured and is shown as a percentage calculated by dividing right side muscle area (mm^2^) with the contralateral area (mm^2^).

### 4.11. Statistical Analysis

For analysis of more than 2 groups with a bigger sample size, parametric one-way Anova test with Tukey post-hoc correction for multiple comparison was used (Figure 3 and Figure 7A). For smaller sample sizes, non-parametric Kruskal-Wallis test with Tukey post-hoc correction was used to compare multiple groups (Figure 4, Figure 5B, Figure 6B,C, Figure 7C and Figure S1). For comparing 2 groups (cells-repair vs. repair-only), non-parametric Mann-Whitney test was used for Figure 8D, Figure 9A–D, Figure 10B,C and Figure S2 and parametric unpaired *t*-test for Figure 10B where Gaussian distribution was assumed based on the endplate area distribution. Endplate frequency distribution was analysed with non-parametric two sample Kolmogorov-Smirnov test (Figure 6D and Figure 10D). Behavioral test results were analysed with two-way Anova with repeated measures in one factor (Figure 9E). The difference was considered to be significant if the *p*-value was less than 0.05. Significant differences were shown with asterisks in the graphs: * *p* < 0.05, ** *p* < 0.01, *** *p* < 0.001, **** *p* < 0.0001. All the results on the graphs are presented as mean ± standard error of the mean (mean ± SEM). All measurements were performed in a blinded fashion so that the analyst was not aware of the sample origin.

## 5. Conclusions

In conclusion, we demonstrated that both spinal cord derived fetal cells and NPCs are able to survive in the injured nerve and can reduce muscle atrophy, while the fetal cells were better than NPCs and lumbar cells were better than thoracic and cervical cells. These cells were able to preserve the muscles both without repair or with 6 weeks delayed nerve repair. Interestingly, injection of different kind of cells was able to keep the muscles at the same stage as with the surgical nerve repair and cell injection together with delayed repair increased the positive effect even more. Therefore, our data supports a novel idea that cell transplantation strategy could save muscles from atrophy and give more time for axonal regeneration, holding a great promise for the future of treatment of peripheral nerve injuries.

## Supplementary Materials

Supplementary materials can be found at www.mdpi.com/1422-0067/18/3/511/s1.

## Abbreviations

ChAT	Choline acetyltransferase
DMEM	Dulbecco’s modified Eagle’s medium
EMG	Electromyography
GFAP	Glial fibrillary acidic protein
GFP	Green fluorescent protein
NMJs	Neuromuscular junctions
NPCs	Neural progenitor cells
P0 cells	Passage zero cells
P2 cells	Second passage cells
α-BTX	α-bungarotoxin

## Appendix A. Grooming Test

In the test, water was poured over animal’s head to elicit grooming movements of the forepaws toward the head. In normal grooming, animal raises both forelimbs, licks them and reaches the area behind the ears. Movement should be bilateral, giving us the opportunity to compare the injured and non-injured side. Grades were given based on the grading scale described in Gu et al. [84]. Shortly, 0—no response, 1—flexion at elbow, not reaching the snout, 2—flexion reaching the snout, 3—reaching below the eyes, 4—reaching to the eyes, 5—reaching to the ears and beyond [84].
