# Supplementary material for: Transplantation of Embryonic Spinal Cord Derived Cells Helps to Prevent Muscle Atrophy after Peripheral Nerve Injury
Source: Int J Mol Sci. 2017 Feb 27;18(3):511. doi: 10.3390/ijms18030511 (PMC5372527; doi:10.3390/ijms18030511)
Supplement: Supplementary file 1 [file ijms-18-00511-s001.pdf]

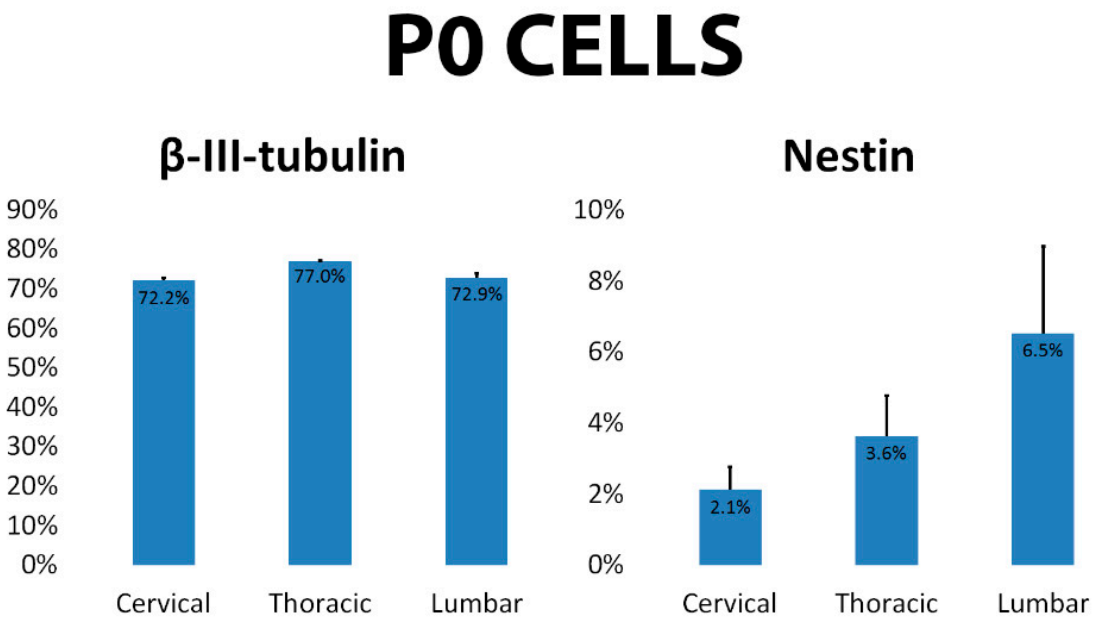

Figure S1. Quantitative in vitro characterization of fetal cells (P0 cells) isolated from different segments of E14.5 embryo spinal cords. Approximately ¾ of the cells expressed neuronal marker III-β-tubulin while no significant differences were seen between the cells from the different spinal cord segments. Number of Nestin+ progenitor cells increased from Cervical < Thoracic < Lumbar though the differences were not significant.

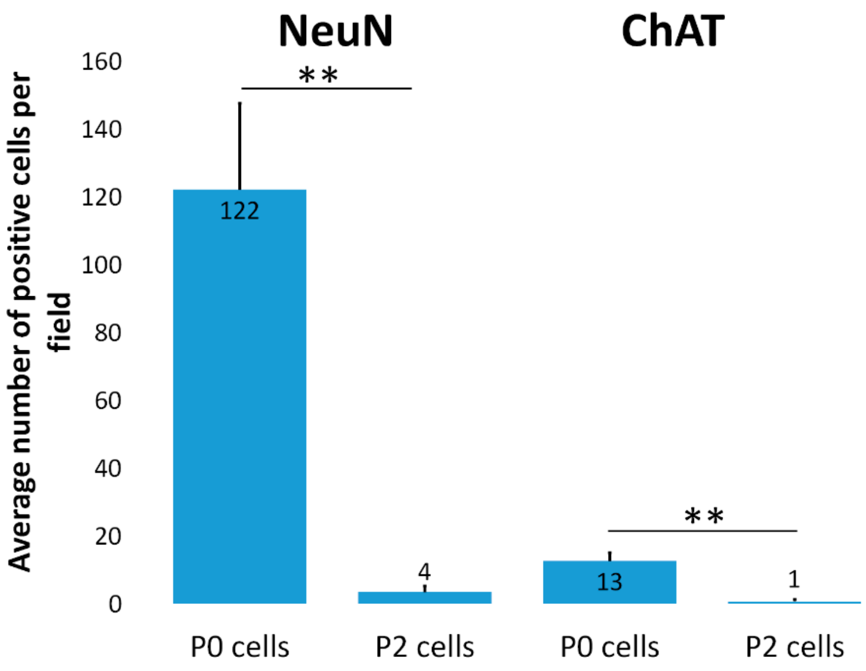

Figure S2. Quantitative characterization of grafted cells in vivo. Number of $NeuN^{+}GFP^{+}$ cells and $ChAT^{+}GFP^{+}$ cells was significantly higher in the P0 cell graft compared to the P2 cell graft. ** $p < 0.001$. Quantification is done for 6 weeks timepoint. n = 5–6 in each group.

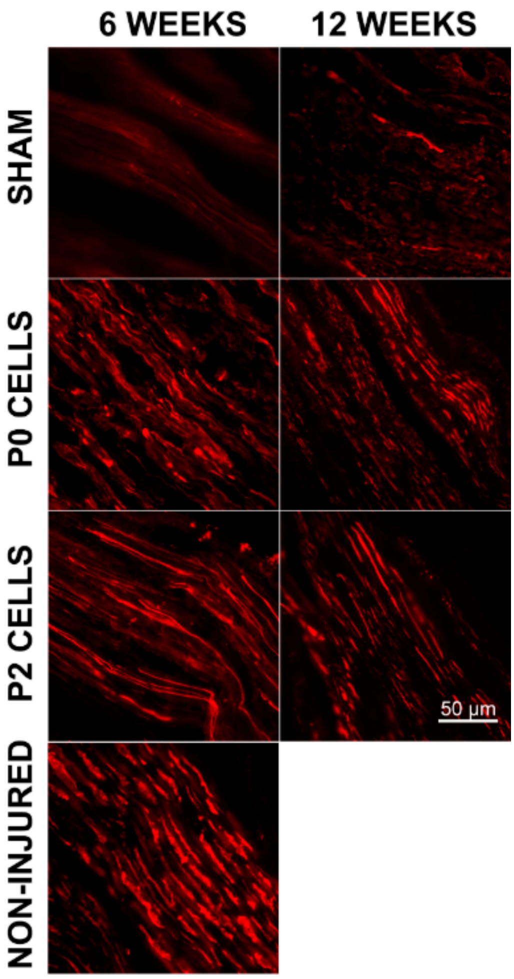

Figure S3. Musculocutaneous nerve longitudinal sections distal to the cell graft showing NF200$^+$ axons 6 and 12 weeks after nerve injury. Very few axons were seen in the sham group while the cell-grafted nerves show numerous thin NF200-positive axons extended towards the target muscle. Thick axons in the non-injured nerve are shown for comparison.
